# Tendon length estimates are influenced by tracking location

**DOI:** 10.1007/s00421-022-04958-8

**Published:** 2022-05-06

**Authors:** Taija Finni, Annamaria Peter, Ra’ad Khair, Neil J. Cronin

**Affiliations:** 1 Faculty of Sport and Health Sciences, Neuromuscular Research Centre, University of Jyväskylä, Viveca 227, Rautpohjankatu 8, 40700 Jyväskylä, Finland; 2 School of Sport & Exercise, University of Gloucestershire, Gloucester, UK

**Keywords:** Achilles tendon, Aponeurosis, Muscle–tendon junction, Rupture, Ultrasound, Strain

## Abstract

**Purpose:**

Measurement of medial gastrocnemius (MG) tendon length using ultrasonography (US) requires the muscle–tendon junction (MTJ) to be located. Previously, the MG MTJ has been tracked from different proximo-distal locations near the MTJ, which could influence estimates of tendon length change due to the different characteristics of the aponeurosis and tendon. We used US to evaluate the effect of tracking point location on MG MTJ displacement during maximal and submaximal (10, 20 and 30% of the non-injured maximal) isometric plantar flexion contractions.

**Methods:**

Displacement behaviour of MTJ was tracked from (1) the exact MTJ; and (2) from an insertion point of a muscle fascicle on the aponeurosis 1.3 ± 0.6 cm proximal to the MTJ, in both limbs of patients with unilateral Achilles tendon rupture (ATR) (*n* = 22, 4 females, 42 ± 9 years, 177 ± 9 cm, 79 ± 10 kg).

**Results:**

In the non-injured limb, displacement (1.3 ± 0.5 cm vs. 1.1 ± 0.6 cm) and strain (6.7 ± 2.8% vs. 5.8 ± 3.3%) during maximal voluntary contraction were larger when tracking a point on the aponeurosis than when tracking the MTJ (both *p* < 0.001). The same was true for all contraction levels, and both limbs.

**Conclusion:**

Tracking a point on the aponeurosis consistently exaggerates estimates of tendon displacement, and the magnitude of this effect is contraction intensity-dependent. When quantifying displacement and strain of the Achilles tendon, the MTJ should be tracked directly, rather than tracking a surrogate point proximal to the MTJ. The latter method includes part of the aponeurosis, which due to its relative compliance, artificially increases estimates of MTJ displacement and strain.

**Supplementary Information:**

The online version contains supplementary material available at 10.1007/s00421-022-04958-8.

## Introduction

Medial gastrocnemius (MG) tendon length is often measured in biomechanical research using ultrasonography (US). The full length of the tendon cannot be captured with current probes, which are limited to 4–10 cm in length, so both ends of the tendon must be imaged separately (e.g. Peltonen et al. 2010) or using the extend of field US imaging (Silbernagel et al. 2016). During an active contraction, the MTJ displaces proximally- elongating the tendon- but also expands in the transverse plane (Hodgson et al. 2006). Thus, measured tendon displacement and strain can vary substantially depending on the position of the US probe over the MTJ (Farris et al. 2013).

In previous studies, the MG MTJ has been tracked from different proximo-distal locations: (1) the most distal part of the muscle, i.e. the MTJ itself (e.g. Arampatzis et al. 2005); or (2) a cross-point between a fascicle and the deep aponeurosis 1–3 cm proximal to the MTJ (Werkhausen et al. 2018). Theoretically, including 1–3 cm of aponeurosis in the tendon length estimate could add extra compliance to the result, depending on aponeurosis stiffness (Arellano et al. 2019; Azizi and Roberts 2009), and this may be more pronounced at high force levels (Arampatzis et al. 2005). Since accurate quantification of tendon behaviour (e.g. strain) requires high measurement precision, it is important to understand how different MTJ tracking methods influence tendon elongation and strain estimates. Moreover, it would be of interest to understand whether changes in muscle–tendon properties, e.g. due to Achilles tendon rupture (ATR), alter the effect of different tracking locations. For example, ATR may lead to an increase in Achilles tendon length after operative (Heikkinen et al. 2017) and conservative management (Khair et al. 2022), a shortening of calf muscle fascicles, and a decrease in aponeurosis stiffness (Peng et al. 2019) in the injured limb. These changes could in turn affect tendon lengthening behaviour during contraction.

In this study, we evaluated the effect of the proximo-distal location of a tracked point on the measured displacement of the MG MTJ during maximal and submaximal isometric plantar flexion contractions. We examined this issue in both legs of unilateral ATR patients, as this also allowed us to determine whether changes in muscle–tendon properties (in the injured leg) alter the effect of tracking point location. We hypothesised that tracking an MG fascicle insertion point just proximal to the MTJ would result in significantly greater displacement than tracking the actual MTJ in both legs. We further expected that this difference would increase with increasing contraction intensity and be smaller in the non-injured versus the injured leg.

## Materials and methods

### Participants

A convenience sample of 22 participants (4 females, *n* = 22, 42 ± 9 years, 177 ± 9 cm, 79 ± 10 kg) gave written consent to voluntarily participate in this study (NoARK, trial registration: NCT03704532). Each individual had experienced an acute Achilles tendon rupture in one of their legs (injured leg, treated non-operatively according to Reito et al. 2018) approximately 1 year (13 ± 2 months) before data collection, while they had no known musculoskeletal problems in the non-injured lower limb. The study protocol was approved by the ethics committee of the Central Finland Hospital District (Approval number: 2U/2018).

### Protocol

Data were collected within one session from both legs. In all cases, the non-injured leg was tested first. While participants lay prone with the foot hanging relaxed over the edge of a plinth, MG tendon resting length was measured using a validated ultrasound-assisted method (Barfod et al. 2015; Intziegianni et al. 2015). Then, in an ankle dynamometer participants performed a warm-up consisting of several submaximal isometric plantar flexion contractions (⁓ 5 min), followed by assessment of maximal voluntary isometric torque (MVC). Participants were instructed to reach the maximum torque in ⁓ 2–3 s, and then to return to the zero level within 2–3 s, resulting in a pyramid-shaped torque curve. Verbal encouragement was given to ensure maximal effort. Slow ramp contractions were then performed to 10%, 20% and 30% of MVC, whereby participants reached the desired torque level in ⁓ 2 s and then returned to the zero level in ⁓ 2 s. For both legs, the target percentages were calculated from the MVC torque of the non-injured leg. During contractions, MG MTJ displacement was recorded with US. For each condition, two successful trials (i.e. target torque level was reached within ± 1%) were recorded and analysed, and the trial showing the largest MTJ displacement was chosen for the final analysis.

### Methodology and analysis

*Torque* Torque was measured with a custom-made ankle dynamometer (University of Jyväskylä, Finland; sampling frequency: 1000 Hz). Participants were seated in the dynamometer with the hip, knee, ankle and first metatarsophalangeal joints fixed at 120°, 0°, 90°, and 0° respectively. To minimise postural changes between trials, the foot was strapped to the dynamometer pedal and the thigh was strapped to the seat just above the knee. To minimise heel lift during contractions, the back of the seat was positioned as close as possible to the pedal. Additionally, a potentiometer under the heel was used to quantify calcaneal displacement during contraction, and confirmed that this value was small even during MVC (mean: 1.0 mm, SD: 1.9 mm, range 0–6.7 mm). Thus, we did not correct tendon displacement values for heel lift, since both of the examined tracking locations should be affected similarly, if at all. A monitor positioned in front of the participants allowed them to follow the torque signal in real-time.

Torque signals were sampled via a 16-bit A/D-board (Power 1401, Cambridge Electronic Design, Cambridge, UK) connected to a personal computer. Digital signals were visualised and recorded in Spike2 Software (Cambridge Electronic Design, Cambridge, UK). To synchronise data acquisition, at the start of each recording, a signal was sent from Spike2 Software to start the video recording in the US device.

*Ultrasonography* B-mode ultrasound data were collected with an Aloka device (Alpha-10; Tokyo, Japan). MG tendon resting length was assessed with a 3.6-cm linear probe (UST-5411, 7.5 MHz), and MG MTJ proximal–distal displacement during isometric contractions was assessed with a 6-cm linear probe (UST-5712, 7.5 MHz). Prior to isometric contractions, the probe was firmly attached to the participants’ leg with an elastic strap. During contractions, ultrasound videos were acquired at 70 Hz for 8 s per trial.

*MG tendon resting length* The tendon insertion on the calcaneus and the most distal point of the MG MTJ were located with US and marked on the skin. The most distal site of MTJ was identified by scanning the area using both longitudinal and transverse views. Both the tendon insertion and origin locations were similarly visible in the non-injured and injured limbs. The distance between these marks, following the curvature of the leg, was then measured with a measuring tape (Barfod et al. 2015). Day-to-day reliability of the tendon length estimates of two assessors was excellent in terms of intraclass-correlation (0.99, 90% CI 0.95–0.99) and standard error of mean (0.17 cm). These reliability tests were done for 20 individuals 2–4 times, with 2–6 days between measurements.

**Fig. 1:**
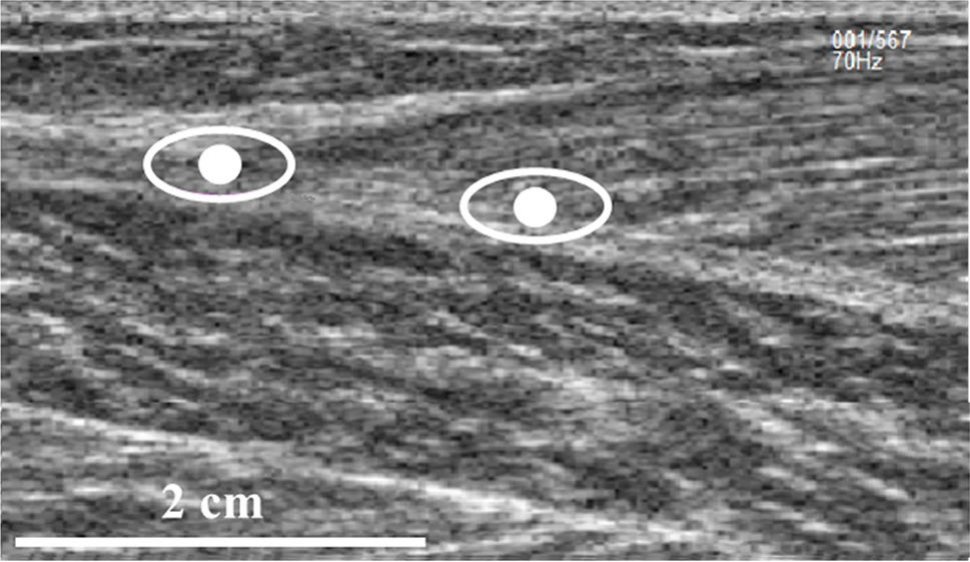
Illustration of the two tracking approaches: (1) the MG MTJ (left ovals, MTJ method); (2) a fascicle intersection with the aponeurosis proximal to the MTJ (right ovals, FAS method)

*MG MTJ displacement tracking* The US probe was attached over the identified MTJ so that during contraction, the area remained visible within the field of view. Correct placement and orientation of the probe was confirmed by observing the image visually during contractions. The position of the MG MTJ was tracked using Tracker 5.1.3 Software (https://physlets.org/tracker/) from two locations: (1) the exact MTJ (MTJ condition); and (2) from an insertion point of a clearly visible fascicle on the aponeurosis 1–3 cm proximal to the MTJ (FAS condition). We used a 4 cm calibration bar and an oval-shaped feature template (0.3–1 cm in length, 0.25–0.50 cm in height; Fig. 1). The FAS was tracked 1.3 ± 0.6 cm proximal to MTJ. This value was added to the MG tendon resting length when calculating tendon strain in FAS condition. Tracking accuracy was visually confirmed for each trial. In case visually perfect accuracy was not reached, the tracking was redone. An example of tracking process is shown in the Supplementary video. The person performing the tracking was blinded to the limb and condition. Reliability of tracking relies on the anatomical location and the template features chosen. We checked that the differences in maximum displacement between two analysers was < 0.1 cm when tracking FAS with the above instruction. Within-analyser reliability had a typical error of 0.06 cm and intraclass correlation of 0.967 [90% CI 0.705–0.997]. Displacement data were smoothed using a 0.14 s moving average.

Maximum proximo-distal MTJ and FAS displacements were extracted from each condition and participant. MG tendon strain was then calculated as $\varepsilon = \frac{\Delta L}{{L0}} \times 100$, where ΔL is the measured displacement and L0 is the resting length. Different resting lengths were used for the MTJ and FAS conditions. Some videos could not be analysed due to video quality. The final sample size for each condition is shown in Table 1.

**Table 1:** Relevant parameters and sample size information for all conditions and each leg separately

Parameter	Non-injured leg	Injured leg
MG resting length (cm)	19.1 ± 1.9	21.6 ± 2.8***
FAS resting length (cm)	20.4 ± 2.1	22.9 ± 3.0***
MVC torque (Nm)	179.6 ± 56.5	130.6 ± 44.9***
Peak displacement FAS (cm)	1.3 ± 0.5¤¤¤	0.8 ± 0.3¤¤¤***
Peak displacement MTJ (cm)	1.1 ± 0.6	0.6 ± 0.3***
Peak Strain FAS (%)	6.7 ± 2.8¤¤	3.7 ± 1.7¤¤***
Peak Strain MTJ (%)	5.8 ± 3.3	3.1 ± 2.0***
Sample sizes		
10% MVC	20	19
20% MVC	17	19
30% MVC	16	19
MVC	16	18

The peak displacements, strains and strain difference were measured during MVC

^*^Difference between limbs, ***p < 0.001

¤Different from MTJ, ¤¤¤*p* < 0.001, ¤¤*p* < 0.01

### Statistical analysis

Statistical analysis was performed using IBM SPSS Software (IBM New York, NY, US). Two-way repeated measures analysis of variance was performed for each leg to assess the interaction between tracking location (MTJ, FAS) and contraction intensity (10, 20, 30, and 100% MVC). Huynh–Feldt correction was applied in case of violated sphericity. In case of an interaction, one-way repeated measures ANOVAs were performed for each variable. Pairwise comparisons were performed after Bonferroni correction. Pairwise *t*-tests were used to characterize differences in selected variables between limbs, as well as between FAS and MTJ (Table 1). Statistical significance level was set at *α* = 0.05 in all cases. Values are reported as mean ± standard deviation in the text. Magnitudes of the differences are also expressed as mean difference ± 95% confidence intervals (95% CI).

## Results

**Fig. 2:**
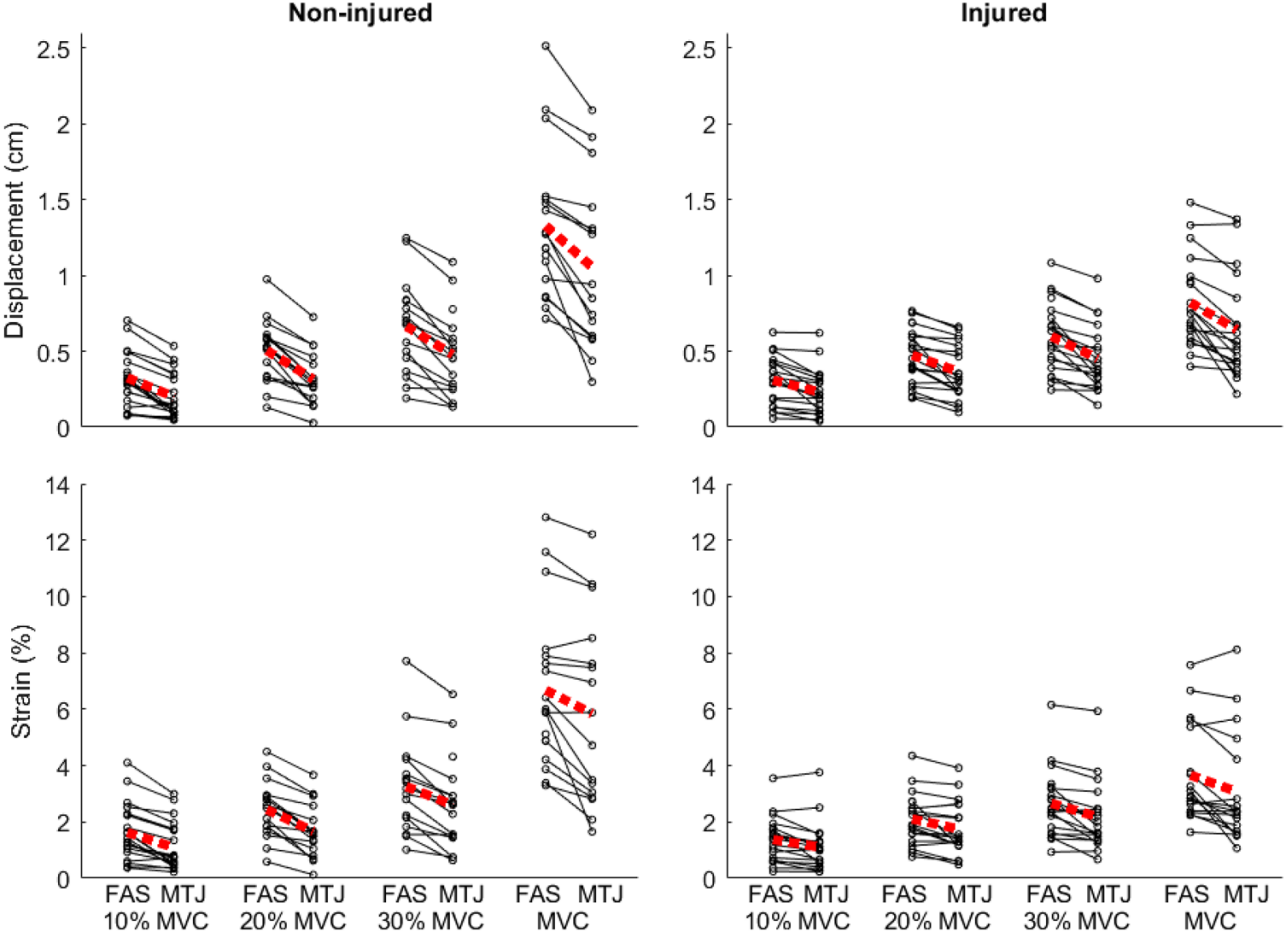
Individual MTJ displacement data for both legs and both tracking locations (i.e. FAS and MTJ) at all contraction intensities. Dashed lines represent group means, and each solid line represents one individual’s data. In all cases, displacement/strain was statistically larger for the FAS condition than the MTJ condition (*p* < 0.05)

Group mean values for MG resting length, MVC torque, and peak MTJ displacement and strain are shown in Table 1. Individual displacement and strain values for all test conditions are shown in Fig. 2.

In the non-injured leg, displacement showed an interaction between tracking location and contraction intensity (*F* = 13.812, *p* < 0.001). There were also main effects of tracking location (*F* = 38.029, *p* < 0.001) and contraction (*F* = 31.841, *p* < 0.001) showing that FAS displacement was always higher than MTJ displacement. This differential displacement increased with contraction level from 0.124 cm [95% CI 0.102–0.146] at 10%–0.294 cm [95% CI 0.238–0.350] at MVC. Strain showed significant interaction (*F* = 5.012, *p* = 0.006), and main effects of location (*F* = 32.781, *p* < 0.001) and contraction were present (*F* = 30.746, *p* > 0.001). The difference in strain between tracking locations increased from 0.5% [0.4–0.6] at 10–1.0% [0.7–1.4] at MVC.

In the injured leg, displacement showed an interaction between tracking location and contraction intensity (*F* = 4.794, *p* = 0.005). There were also main effects of tracking location (*F* = 50.572, *p* < 0.001) and contraction intensity (*F* = 38.025, *p* < 0.001), whereby FAS displacement was higher than MTJ displacement at all contraction levels. The differential displacement between tracking locations increased with contraction intensity from 0.074 cm [0.040–0.107] at 10% to 0.170 cm [0.107–0.232] at MVC. Strain showed no interaction but main effects of location (*F* = 21.182, *p* < 0.001) and contraction were present (*F* = 29.125, *p* > 0.001). On average, the strain was 0.4% [0.3–0.5] greater when tracking FAS than MTJ with significant differences between all contraction levels (*p* < 0.001).

## Discussion

We examined the effect of tracking MTJ displacement using two different approaches: tracking the actual MTJ, or a point 1.3 cm proximal to the MTJ. We found that displacement and strain values were statistically larger when tracking a point on the aponeurosis than when tracking the MTJ itself. The difference in displacement between the MTJ and FAS tracking locations increased with contraction intensity in both limbs. Regarding strain, the difference between MTJ and FAS increased with contraction intensity in the non-injured limb, but in the injured limb the interaction of tracking location and contraction level was not statistically significant, albeit showing a similar trend. Overall, tracking a point on the aponeurosis, even just 1–3 cm proximal to the MTJ, exaggerates estimates of tendon displacement and strain.

Previous studies have shown that during MVC, aponeurosis elongation exceeds that of the Achilles tendon (Arampatzis et al. 2005; Finni and Komi 2002; Kubo et al. 2002). This is possible since the aponeurosis and tendon have different mechanical properties (Lieber et al. 1991). While the tendon is stiffer and follows a curvilinear force–elongation pattern, the aponeurosis behavior is more complex (Azizi and Roberts 2009). Several studies have shown that upon contraction, aponeurosis may not behave uniformly and may strain less than tendon (Finni et al. 2003; Lieber et al. 2000; Zuurbier et al. 1994). While aponeurosis displacement may be variable during active contraction, the present results suggest that at least the very distal aponeurosis of medial gastrocnemius muscle can add extra compliance to estimates of tendon displacement and strain.

We found that the choice of tracking location led to small but statistically significant differences in displacement and strain. During MVC, the differences were 0.17 cm and 0.6% in the injured limb and 0.30 cm and 1.0% in the non-injured limb. Regarding displacement, the result corroborates the findings of Arampatzis et al. (2005) who reported that the difference between tendon and aponeurosis elongation increased with contraction level. The present mean strain of 5.8% assessed using tracking of MTJ in the non-injured limb during MVC is in concordance with previous studies; ultrasound-based estimates of Achilles tendon strain in healthy adults during MVC generally range between about 4.4% and 6.5% (Arampatzis et al. 2005, 2008; Farris et al. 2013; Magnusson et al. 2001). Thus, the differences in strain estimates between the FAS and MTJ methods reported here are substantial (19% and 17% in injured and non-injured limb, respectively). Importantly, the differences also vary with torque level, meaning that the resulting error when using the FAS method is not systematic but increases with contraction level. It follows that the FAS approach should be avoided when aiming to determine the behaviour of the Achilles tendon only.

As expected, in the injured limb of ATR patients, we found much lower MVC torque and lower maximal displacement and strain compared to the non-injured leg. Nonetheless, the same observation of larger displacement in FAS than MTJ in both limbs confirms that even in cases where the properties and interaction behaviour of the muscle-aponeurosis-tendon system are dramatically altered (Khair et al. 2022; Peng et al. 2019), the FAS method still results in exaggerated estimates of MTJ displacement. Regarding strain, there was no statistically significant interaction between tracking location and contraction level, suggesting that the difference in strain does not depend on contraction intensity. Although the mean values showed an increasing trend (0.2% at 10%, 0.3 at 20%, 0.4% at 30% and 0.6% at MVC), the lack of statistical significance may be due to the fact that strain values in the injured limb were much lower than in the non-injured limb.

It may be argued that the non-injured limb of ATR patients does not represent a healthy tendon since there is a risk of contralateral rupture; in one study 8.5% of ATR patients experienced a contralateral rupture (Palma et al. 2018). However, in the present study we focused on MG MTJ displacement and strain, and we found similar results to those typically reported in individuals who have never suffered an ATR. Furthermore, we previously found in the same patient cohort that while the injured limb had lower plantarflexion strength and a longer tendon length, stiffness was not different between limbs (Khair et al. 2022). As a limitation it should be noted that we examined proximal–distal displacement and strain in two dimensions, although muscle–tendon junction shape may change 3-dimensionally (Farris et al. 2013) and rotate laterally along with the muscle (Hodgson et al. 2006). While lateral shifts could slightly modify the results, movements in the proximal–distal direction dominate and are potentially the major contributor to the measurement errors of tendon displacement and strain.

## Conclusions

When quantifying displacement and strain of the Achilles tendon, the MTJ should be tracked directly, rather than tracking a surrogate point proximal to the MTJ. The present study showed that due to its relative compliance, including only 1–3 cm of aponeurosis to the measure of medial gastrocnemius tendon length artificially increases estimates of MTJ displacement and strain.

## Supplementary Information

Below is the link to the electronic supplementary material.

Supplementary file1 (MP4 40452 KB)

## Data Availability

The datasets generated and analysed during the current study are available from the corresponding author on reasonable request.

## Abbreviations

ATR: Achilles tendon rupture
CI: Confidence interval
FAS: Fascicle on the aponeurosis 1–3 cm proximal to the MTJ
MG: Medial gastrocnemius
MTJ: Muscle–tendon junction
MVC: Maximal voluntary isometric torque
US: Ultrasonography
